# Saliva Crystallization Occurs in Female Bornean Orangutans (*Pongo pygmaeus*): Could It Be a New Option for Monitoring of Menstrual Cycle in Captive Great Apes?

**DOI:** 10.1371/journal.pone.0159960

**Published:** 2016-07-26

**Authors:** Anna Kubátová, Tamara Fedorova

**Affiliations:** Department of Animal Science and Food Processing, Czech University of Life Sciences Prague, Prague, Czech Republic; University of Rouen, France, FRANCE

## Abstract

Saliva crystallization was previously studied in both humans and animals with various results. The study aimed to confirm of the presence of saliva crystallization in female Bornean orangutans (*Pongo pygmaeus*), to evaluate the quality of samples which were collected from animals and processed by keepers, and to test preliminarily if the saliva crystallization could be connected with menstrual cycle and could serve as a cheap, quick and simple method for the basic monitoring of their reproductive status. The research was carried out from September 2014 to January 2015. Sampling of saliva was done in three female orangutans from three zoological gardens (Dvur Kralove, Usti nad Labem, Bojnice) daily, mostly by tongue prints on glass slides with ground edges or by sampling directly from the mouth using plastic spoons from which the saliva was transferred onto glass slides. Samples were evaluated by light microscopy with ×400 magnification. The quality of the sample and type of crystallization was assessed for two different approaches. In total, 246 samples were evaluated. We confirmed the presence of saliva crystallization in orangutans. The quality of samples was variable however acceptable. Unfortunately, it was impossible to detect exact fertile period in two females. However in one orangutan female, when the crystallization was evaluated by the approach typically used in humans, we discovered that saliva crystallization during the fertile period significantly differed from saliva crystallization in the non-fertile period. This points out the possibility of using saliva crystallization for detection of the fertile period in orangutans. However, further research was recommended.

## Introduction

Bornean orangutans (*Pongo pygmaeus*) are considered an endangered species with a decreasing population trend [1]. Unfortunately, their reproduction is the slowest among great apes [2, 3]. Especially in *ex situ* conservation programs, which mainly take place in zoos, there is an increasing need for proper reproduction management, including methods of ovulation and pregnancy detection, which help these programs to succeed [4]. In some zoos, male and female orangutans are kept separately and put together for mating purposes only. Especially in these situations, information about the fertile days of females is necessary.

It is possible to find similarities in reproduction of orangutans and humans [5, 6]. Female orangutans have a menstrual cycle which lasts for around 27–28 days [3, 7] as in humans [8]. In women, the fertile period occurs circa 5–6 days before and during ovulation [9], which occurs around the 13^th^ or 14^th^ day of the cycle [10] similar as in orangutans [11]. The menstrual cycle of orangutan female can be sometimes monitored by visible menstruation. However, it is not as common and the presence of blood can often only be detected by testing urine [3, 12]. Also, the presence of blood in urine does not always mean the menstruation time. Captive male orangutans are known for inducing forced copulations irrespective of female resistance even on a daily basis [13]. After such mating, females can receive soft tissue injuries that can result to bleeding [14].

Moreover, the length of the menstrual cycle is variable both among individual females and among individual cycles of concrete female [12] and the ovulation time can also differ individually [15]. Because of this, fertile day period is just approximated by urine testing. Therefore, an easier method for the detection of fertile days is needed.

Crystallization of body fluids, related to menstrual cycle, was first observed in human cervical mucus 60 years ago [16]. Saliva crystallization was later also described in humans in association with the fertile period [10, 17–19] and pregnancy detection [20]. Saliva crystallization changes during the menstrual cycle. According to certain authors [10, 21, 22], it is caused by cyclic changes in hormonal levels, concretely estrogens. Estrogens have been found to be a cause of rising of electrolytes, which are necessary for the formation of crystals. Fern-like crystallization is typical for estrus [17, 19, 23]. Certain authors admit that saliva crystallization has potential in fertile period monitoring [10, 17–20, 24] and pregnancy detection [20, 25] in humans. However, there are also studies that disprove these information [26, 27].

As in humans, saliva crystallization in association with reproductive status monitoring and optimal mating time determination was studied in females of beagles (*Canis familiaris*) [28], Holstein Friesian cattle (*Bos taurus*) [29] and Bactrian camels (*Camelus bactrianus*) [30]. In beagles, saliva crystallization developed just during the follicular phase of the cycle (proestrus and estrus) and changed step by step from partial to total crystallization. It occurred few days before and after optimal mating time. Total crystallization was synchronized with maximal estrogen levels, however authors of the study recommended saliva crystallization only as supplementary method to others [28].

Monitoring of reproduction by non-invasive methods and animal welfare belong to the essential issues for breeding of endangered species, including primates [31]. Collection of saliva is done directly from animal mouth, but it is commonly considered as a non-invasive method of collection [29]. Hormone analyses from saliva has been already done in non-human primates (e.g. studies of Kutsukake et al. [32], or Wobber et al. [33] and others); however, these studies have mostly focused on the monitoring of stress [31] and saliva was successfully used for menstrual cycle monitoring only in capuchin monkeys (*Cebus apella*) [34]. To the best of our knowledge, saliva crystallization, as a quick and cheap non-invasive method for monitoring fertile periods with simply interpretable results [28, 29], has never been studied in non-human primates or great apes even though they are closely related to humans [35, 36].

For saliva crystallization, samples of saliva can be obtained by different methods, e.g. using tampons [10, 17, 21], fingers [19, 28] or other devices [37] and after collection, saliva should be smeared on a glass slide [28, 29]. Sampling can be also performed directly by pressing the tongue on a glass slide [28]. Samples are left to dry at room temperature [17, 19, 20, 38] and evaluated for crystallization.

Crystallization can be classified by different methods. The first approach is mostly used in studies in humans and distinguishes whether the crystallization is typical for non-fertile days, the transit period, or fertile days [17–19, 23]. The second approach, used e.g. in cattle [29] and Bactrian camels [30], distinguishes four main types of saliva crystallization—none, branch-like, fir-like, and fern-like, as well as their combinations.

As great apes are closely related to humans [35, 39], we hypothesized that saliva crystallization will occur in orangutans and the method will be applicable as in humans. The aims of this study were 1) to prove the existence of saliva crystallization in female Bornean orangutans; 2) to evaluate the quality of samples which were collected from animals and processed by keepers; 3) to test preliminarily if the saliva crystallization could be connected with menstrual cycle and serve in the future as a cheap, quick and simple method for basic monitoring of menstrual cycle in great apes.

## Materials and Methods

### Ethic Statement

The research was carried out in accordance with the current legislation of the Czech Republic and the European Union. The research was approved by the management of all of the zoological gardens involved, i.e. Zoo Dvur Kralove, Zoologicka zahrada Usti nad Labem and Zoologicka zahrada Bojnice. According to the Act no. 246/1992 Coll., on the protection of animals against cruelty of the Czech Republic and Directive 2010/63/EU of the European Parliament and of the Council of 22 September 2010 on the protection of animals used for scientific purposes, the study met all of the requirements of non-invasive research so no other ethical approval was needed. The sampling of saliva was carried out by the zookeepers, which take a regular care of the animals, as a part of training or enrichment. Animals were not forced into the cooperation and did not suffer stress. They were accustomed to the sampling devices and sampling process step by step from the beginning of the research period.

### Animals and Management

Saliva was sampled in three non-pregnant Bornean orangutan females (*Pongo pygmaeus*) from three zoological gardens that are members of the European Association of Zoos and Aquaria (EAZA)—Zoo Dvur Kralove (n = 1) and Zoologicka zahrada Usti nad Labem (n = 1) in the Czech Republic and Zoologicka zahrada Bojnice (n = 1) in the Slovak Republic. All females were already sexually matured, had offspring in the past and all of them took care of their last young one during the research period. See Table 1 for details.

**Table 1:** Detailed information about females included in the research.

Breeding facility	Orangutan female	Date of birth	Date of birth of the last young one
Bojnice	Nanga	29. 1. 1995	18.5.2009
Dvur Kralove	Žaneta	29. 6. 1976	13.11.2010
Usti nad Labem	Ňuninka	1987	17.12.2011

In Dvur Kralove, there was no male, so the female was housed just with her last offspring. In Usti nad Labem, female was always together with her two last offspring. They were accompanied by male in the morning and afternoon, at night they stayed separately. In Bojnice, male, female and their last offspring were housed together only during morning hours and in the afternoon and at night female and the offspring were separated from the male. When the female was supposed to be in estrus, she was housed together with the male continuously.

The equipment of housing facilities was very similar across zoos and meet requirement of EAZA. Outside paddock was available in all cases. Light regime was always natural so no artificial changes were done. Regular enrichment activities were mostly based on providing of branches, bark or wood-wool and animals had the opportunity to sleep in hammocks. The diet of animals consisted mainly from vegetables and fruits and it was supplemented by eggs, meat, dairy products or other types of feed. The feed was provided in smaller amounts and regularly, mostly four times a day. Water was always provided *ad libitum*.

### Monitoring of Reproduction

Potential pregnancy was monitored in females in Bojnice and Usti nad Labem. For this purpose, their urine was tested by the pregnancy test GS Mamatest 10 (Green-Swan Pharmaceuticals CR, a.s.) monthly. All results were negative. The female in Dvur Kralove was separated from male for the period longer than gestation and so none of females included in the study was pregnant.

The menstrual cycle was only monitored in the female in Bojnice, because this female had a regular cycle with visible menstruation that was recorded. During the five month research period, menstruation was observed three times and the bleeding was visible for 1–2 days. The first day of bleeding was considered the first day of the cycle. The average length of menstrual cycle was 28 days. According to studies of reproduction in humans [10, 12] and orangutans [11], 13^th^-14^th^ day of the cycle was approximated as an ovulation day and because the actual reproductive state of the female was only approximated, a longer time period was considered for fertile days, which was consistent with the 8^th^-19^th^ days of the cycle [40]. The rest of the cycle was considered non-fertile days. In females in Dvur Kralove and Usti nad Labem, visible menstruation was not observed during the research period and no other monitoring of the menstrual cycle was practiced.

### Collection of Samples

Samples of saliva were collected daily by zookeepers in following periods: September-November 2014 in Dvur Kralove, September-December 2014 in Usti nad Labem, and from September 2014 till January 2015 in Bojnice. The keepers underwent the basic training and received the instructions how to collect saliva, make smears and store the samples. After that they performed all these steps independently.

In Dvur Kralove, sampling was done using glass slides with ground edges that were used for tongue prints or scraping of saliva from the inner part of lips. In Bojnice, saliva was collected by plastic spoons from the tongue or inner part of the lips. Because the female in Usti nad Labem was untamed, spits from the ground were mainly collected. Exceptionally, licking of ground edged glass slides, plastic spoons and cotton swabs was used there.

In all breeding facilities, sampling was done between major feeding times, however no exact time interval between feeding and sample collection was determined. The sampling procedure was very irregular in the untamed female in Usti nad Labem. In Dvur Kralove and Bojnice, sampling was done usually in morning hours. In Bojnice, feed was used to attract female’s attention to come closer for sampling and also as a reward during sampling. In spite of this in Dvur Kralove, no feed was provided during sampling.

Immediately after sampling, saliva was transferred and spread onto a glass slide, if the sampling was not done directly by it. Then, the glass slide was marked with the date of sampling, left to dry at room temperature for circa 15 minutes and transferred to a box case for the storage and transport of samples. With approximately one month intervals, boxes were transported to the laboratory of the Department of Animal Science and Food Processing, Czech University of Life Sciences (CULS) Prague. There, samples were evaluated.

### Evaluation of Crystallization

**Fig 1:**
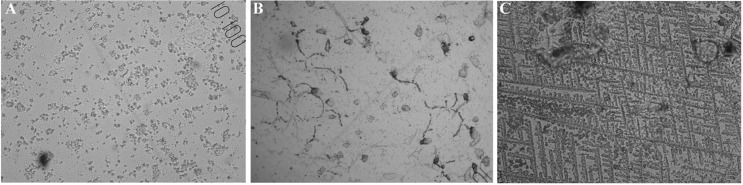
Crystallization typical for non-fertile days (A), transit period (B) and fertile days (C) (×400 magnification).

Samples were evaluated by light microscope with a magnification of ×400 by one well trained researcher. Crystallization was classified by two different methods. First method (NTF scale), normally used in humans [17–19, 23], was classifying the crystallization with the connection to the estrus cycle. Three types of crystallization were distinguished—crystals typical for the non-fertile days (N), the transit period (T), or the fertile days (F). Crystallization was evaluated as typical for the non-fertile days when no crystallization or small random non-compact crystals in the form of discrete dots were visible. The samples evaluated as from the transit period were characterized by intermediate crystallization created by a combination of dots and other types of crystallization, like branch-like, fir-like and fern-like (see the description below). Completely formed ferning pattern had to be found to evaluate the samples as from the fertile days period [17–19, 23] (see Fig 1).

**Fig 2:**
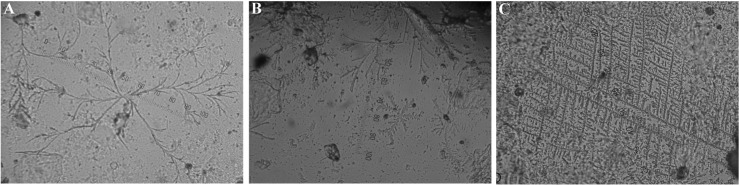
Branch-like (A), fir-like (B) and fern-like (C) crystallization (×400 magnification).

The second method, normally used in animals [29, 30], was more detailed and distinguished four main types of saliva crystallization—none (0), branch-like (BL), fir-like (FIL), and fern-like (FEL) (see Fig 2) and their combinations—branch-like and fir-like (BL+FIL), branch-like and fern-like (BL+FEL), fir-like and fern-like (FIL+FEL), branch-like and fir-like and fern-like (BL+FIL+FEL). We called this method as the branch-, fir- and fern-like scale (BFF scale). For BL crystallization evaluation, small random non-compact crystals or crystals in shape of contorted and irregular twigs were typical. Samples with crystals in the shape of fir branches (*Abies* sp.) were considered as FIL crystallization. As FEL crystallization, samples were evaluated when showing crystals with straight branches creating (nearly) right angles like in ferns (e.g. *Dryopteris* sp.).

In all samples, type of crystallization by BFF and NTF scales were assessed. Every sample was classified by one final type of crystallization for both scales; when more types of crystallization occurred in one sample, the predominant type of crystallization was used. The quality of the sample was considered good when it was possible to evaluate the sample without problems, or bad when the sample was visibly contaminated (e.g. by feedstuff) or when the layer of saliva was too thin or too thick to distinguish crystals.

### Data Analysis

Collected data were statistically evaluated in the program Statistica Cz 12 (StatSoft, Inc., 2013). For all calculations, a significance level α = 0.05 was established and all calculated numerical values were rounded off to two decimal places. To analyze the data, frequency tables, contingency tables and Pearson’s chi-squared tests were used.

The results of saliva crystallization of the female from Bojnice were retrospectively compared with her menstrual cycle monitoring recordings.

## Results and Discussion

### Saliva Collection and Sample Quality

In total, 246 saliva samples from three non-pregnant Bornean orangutan females were collected and evaluated. All data are included in S1 File. We confirmed the presence of saliva crystallization in orangutans. The quality of samples was significantly different among zoos (P < 0.01), see Table 2. However, in average 88.21% of samples had a good quality and were useable for evaluation of saliva crystallization. None of the samples were too thin.

**Table 2:** Collected samples and their quality.

Zoo	No. of collected samples	Frequency (%) of samples which had		
		good quality	contamination	too thick saliva smear
Usti nad Labem	11	100.00 (n = 11)	0.00 (n = 0)	0.00 (n = 0)
Dvur Kralove	94	95.74 (n = 90)	0.00 (n = 0)	4.26 (n = 4)
Bojnice	141	82.27 (n = 116)	17.02 (n = 24)	0.71 (n = 1)
**In total**	**246**	**88.21** (n = 217)	**9.76 (n = 24)**	**2.03 (n = 5)**

Orangutan females from zoos in Dvur Kralove and Bojnice became used to the sampling process very quickly, meaning that regular daily sampling was possible. In contrast, there were problems with the sampling in the female from Usti nad Labem. No progress in training was observed and she did not get used to the sampling process, so the number of samples from this female was very low. In agreement with the studies of Kutsukake et al. [32] and Behringer et al. [41], the females were accustomed to the sampling devices and sampling process step by step from the beginning of the research period to prevent stress in these animals and to preserve the non-invasiveness of this method. Stress was also minimized by using a sampling method provided by zookeepers themselves; thus, there was no need to introduce the females to new individuals.

The differences in quality of samples across zoos could be caused by different way of sampling technique, however no publication concerning this topic was found. Also we must consider that samples were processed by different keepers. A higher level of contaminated samples (almost 18%) in the female from Bojnice was probably caused by sampling too closely after feeding time, by providing feed-like rewards during sampling or by chewing of ever-present branches, bark or wood-wool before sample collections. The contamination of samples should be eliminated and contaminated samples should not be evaluated, because contamination can influence the way in which saliva crystallizes [18, 19].

### Saliva Crystallization

In general, 70.51% (N = 153) samples of good quality crystallized. While crystallization types evaluated by the NTF scale (Table 3) significantly differed among females (P = 0.01), crystallization types assessed by the BFF scale (Table 4) did not significantly differ among them (P > 0.05). The most common crystallization type according to the BFF scale in female Bornean orangutans was simple BL, which corresponds to the results in Bactrian camels [30] or domestic cattle [29]. Other types of crystallization according to the BFF scale were significantly less common and the results did not resemble those in any other animals.

**Table 3:** Occurrence of crystallization types by the NTF scale in all 217 good quality samples.

Zoo	Crystallization type typical for (%)		
	non-fertile days (N)	transit period (T)	fertile days (F)
Usti nad Labem	90.91 (n = 10)	9.09 (n = 1)	0.00 (n = 0)
Dvur Kralove	46.67 (n = 42)	43.33 (n = 39)	10.00 (n = 9)
Bojnice	38.79 (n = 45)	54.31 (n = 63)	6.90 (n = 8)

**Table 4:** Occurrence of crystallization types by the BFF scale in all 217 good quality samples.

Crystallization type	Number of samples	Relative frequency (%)
BL	103	47.47
0	64	29.49
FIL	16	7.37
BL+FEL	12	5.53
BL+FIL	10	4.61
FEL	8	3.69
FIL+FEL	2	0.92
BL+FIL+FEL	2	0.92

Legend of crystallization types: 0—none, BL—branch-like, FIL—fir-like, FEL—fern-like, BL+FIL—branch-like and fir-like, BL+FEL—branch-like and fern-like, FIL+FEL—fir-like and fern-like, BL+FIL+FEL—branch-like, fir-like and fern-like.

Medially developed crystallization (i.e. combination of dots and other types of crystallization), which should be typical for the transit period, occurred during the entire cycle with almost the same frequency on fertile and non-fertile days. Nevertheless, the occurrence of transitional crystallization during the entire menstrual cycle was also observed in women’s saliva by Berardono et al. [27].

In this study, the samples were evaluated by experienced researcher as in blind experiment, i.e. the evaluator did not know the period of the cycle in which the samples were collected. However, after basic training, the crystallization can be easily observed and evaluated by keepers themselves. This is big advantage of saliva crystallization because keepers can receive immediately the information about reproductive status of animals. Nevertheless, this subjectivity in the process of evaluation is a strong negative of this method. However, even this can be eliminated by the usage of higher numbers of informed but independent evaluators whose partial results will be connected to the final conclusion.

In the female from Usti nad Labem with 3 years old young one, we cannot exclude that the results were not affected by lactation amenorrhea, because lactation amenorrhea last from 3 to 6 years in orangutans [42]. We did not note any crystallization typical for fertile days in this female. However, other types of crystallization were found in this female which corresponds with findings in postpartum women [20]. In other two females, influence of lactation amenorrhea can be excluded, because the average inter birth interval in zoo kept orangutans is around 5 years [3, 43, 44] and covers also the length of pregnancy, which vary between 223–275 days [3]. This means that the majority of females are cycling already 4 years after parturition. Also, in both these females, crystallization typical for fertile days was observed.

The fact that there were differences among the females by the NTF scale but not by the BFF scale was probably caused by the difference in these methods. The NTF scale is directly connected with evaluation of reproduction and considers the reproductive state of animals, which differed among females in the study–one of them was probably in lactation amenorrhea.

### Possible Connection with Menstrual Cycle

**Fig 3:**
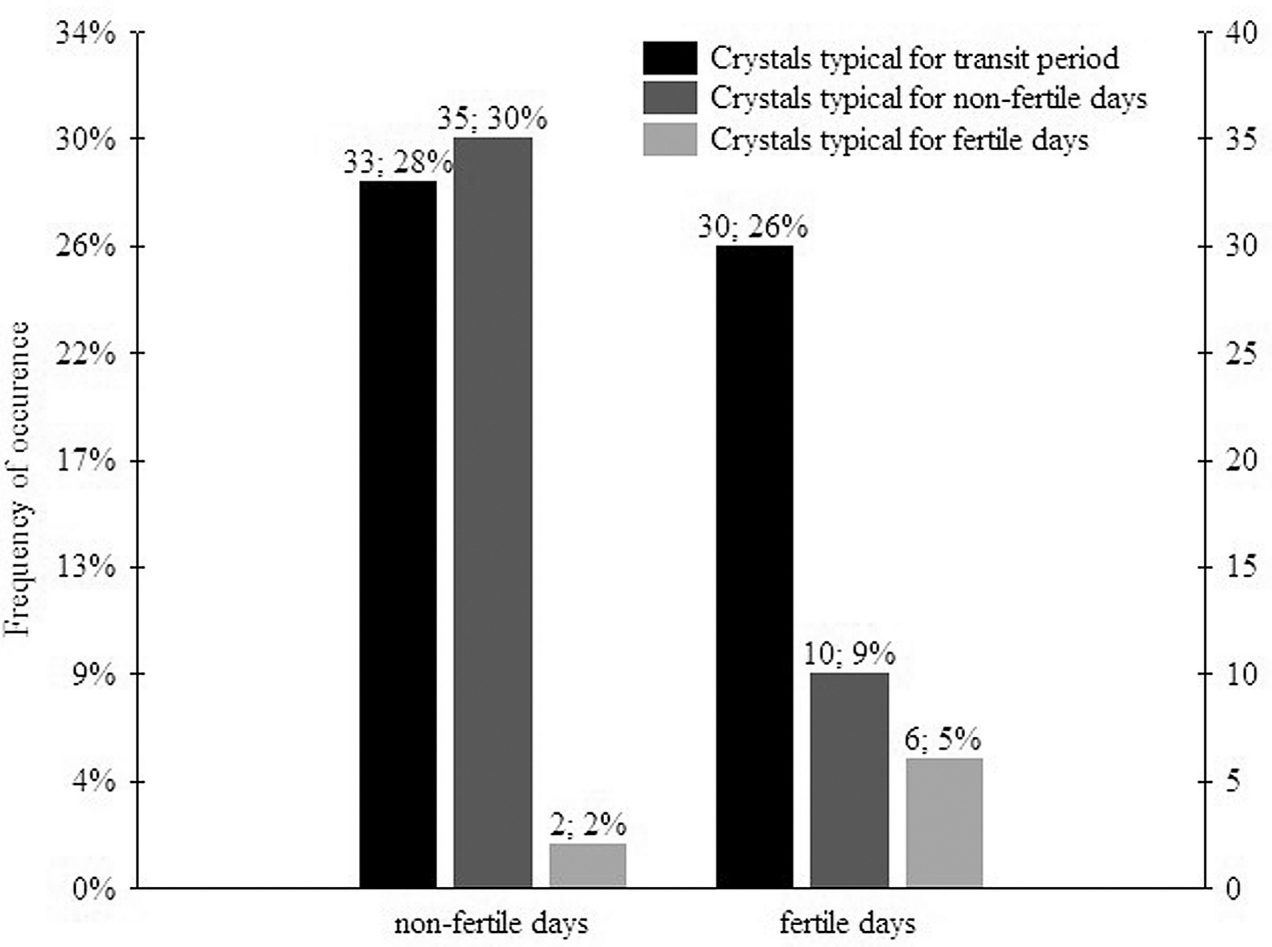
Frequencies of the occurrence of different types of crystallization by the NTF scale during the fertile and non-fertile days in the female from Bojnice. A significant difference in types of saliva crystallization collected in fertile and non-fertile days of the cycle was found (Pearson’s chi-squared test, P < 0.01).

A significant difference in types of saliva crystallization evaluated by the NTF scale between samples collected in fertile and non-fertile days of the cycle was found in the female from Bojnice (P < 0.01). Frequencies of the occurrence of different types of crystallization by the NTF scale during the fertile and non-fertile days are provided in Fig 3. However, types of saliva crystallization evaluated by the BFF scale did not significantly differ between samples from fertile and non-fertile periods of the cycle (P > 0.05). NTF scale scoring worked in orangutans probably because this method was developed directly for humans that are closely related to them [35, 36]. In spite of this, connection between the saliva crystallization scored by the BFF scale and the reproductive status of females was proved just in cattle [29].

The fact that types of crystallization according to the NTF scale differed between fertile and non-fertile days in the female from Bojnice suggests that the saliva crystallization changes during the menstrual cycle of orangutan females, similar as in dogs [28] and humans [17, 19]. Crystals typical for non-fertile days were more common in the non-fertile period of the cycle. In spite of this, the crystallization indicating the fertile days occurred more in the fertile day period or close to this time interval. This corresponds with the results obtained in women [17–19, 23] and suggests the possibility to use saliva crystallization for fertile day monitoring in Bornean orangutans and maybe also in other non-human primates.

We must also consider the possibilities that the length of menstrual cycle can fluctuate among various cycles in one female [12] and that even though orangutans are very close to humans [35, 39] there are still some differences. No matter how promising these results seem, the samples for these reproduction-connected analyses were collected from a single female Bornean orangutan whose actual reproductive status at the time of sampling was only approximated. Before concluding, preliminary results should be verified in more females. However, saliva crystallization seems to be a possible method for fertile days monitoring in Bornean orangutans when supplemented with others, e.g. menstruation recordings, as in humans [17–19, 22] and dogs [28]. Testing this method on other primate species is also possible.

## Supporting Information

S1 File The file containing the data used in this study. (XLSX)

## References

[1] Ancrenaz M, Marshall A, Goossens B, van Schaik C, Sugardjito J, Gumal M, et al. The IUCN Red List of Threatened Species: *Pongo pygmaeus*: IUCN; 2008 [2015-02-14]. Available from: http://www.iucnredlist.org/.

[2] Wich SA, Utami-Atmoko SS, Setia TM, Rijksen HD, Schürmann C, van Hooff JA, et al Life history of wild Sumatran orangutans (*Pongo abelii*). Journal of Human Evolution. 2004;47:385–98. 15566945 10.1016/j.jhevol.2004.08.006

[3] Shumaker RW, Wich SA, Perkins L. Reproductive life history traits of female orangutans (*Pongo* spp.). Interdisciplinary Topics in Gerontology. 2008;36:147–61. 10.1159/000137705 18523377

[4] Swaisgood RR, Zhou X, Zhang G, Lindburg DG, Zhang H. Application of Behavioral Knowledge to Conservation in the Giant Panda. International Journal of Comparative Psychology. 2003;16:65–84.

[5] Collins DC, Graham CE, Preedy JR. Identification and measurement of urinary estrone, estradiol-17 beta, estriol, pregnanediol and androsterone during the menstrual cycle of the orangutan. Endocrinology. 1975;96:93–101. 162882 10.1210/endo-96-1-93

[6] Graham C. Reproductive Physiology In: Schwartz JH, editor. Orang-utan Biology. New York: Oxford University Press; 1988 p. 383.

[7] Shimizu K, Udono T, Tanaka C, Narushima E, Yoshihara M, Takeda M, et al Comparative study of urinary reproductive hormones in great apes. Primates. 2003;44:183–90. 12687484 10.1007/s10329-002-0021-9

[8] La Marca A, Stabile G, Carducci Artenisio A, Volpe A. Serum anti-Mullerian hormone throughout the human menstrual cycle. Human Reproduction. 2006;21:3103–7. 16923748 10.1093/humrep/del291

[9] Wilcox AJ, Weinberg CR, Baird DD. Timing of sexual intercourse in relation to ovulation. Effects on the probability of conception, survival of the pregnancy, and sex of the baby. The New England Journal of Medicine. 1995;333:1517–21. 7477165 10.1056/NEJM199512073332301

[10] Alagendran S, Archunan G, Prabhu SV, Orozco BE, Guzman RG. Biochemical evaluation in human saliva with special reference to ovulation detection. Indian Journal of Dental Research. 2010;21:165–8. 10.4103/0970-9290.66625 20657081

[11] Nadler RD. Sexual Behavior of Orangutans (*Pongo pygmaeus*) In: Nadler RD, Galdikas BFM, Sheeran LK, Rosen N, editors. The Neglected Ape. Boston, MA: Springer US; 1995 p. 223–37.

[12] Graham C. Reproductive Biology of the Great Apes: Comparative and Biomedical Perspectives New York: Academic Press; 1981. 456 p.

[13] Nadler RD. Sexual behavior of captive orangutans. Archives of sexual behavior. 1977;6:457–75. 563223 10.1007/BF01541151

[14] Muller MN, Wrangham RW, editors. Sexual coercion in primates and humans: an evolutionary perspective on male aggression against females Cambridge: Harvard University Press; 2009.

[15] Wilcox AJ, Dunson D, Baird DD. The timing of the “fertile window” in the menstrual cycle: day specific estimates from a prospective study. BMJ. 2000;321:1259–62. 11082086 10.1136/bmj.321.7271.1259 PMC27529

[16] Papanicolaou GN. A general survey of the vaginal smear and its use in research and diagnosis. American Journal of Obstetrics & Gynecology. 1946;51:316–28. 21017069 10.1016/s0002-9378(16)40012-8

[17] Barbato M, Pandolfi A, Guida M. A new diagnostic aid for natural family planning. Advances in Contraception. 1993;9:335–40. 8147248 10.1007/BF01983212

[18] Galati G, Trapani E, Yacoub M, Toccaceli MR, Galati GM, Fiorelli C, et al A new test for human female ovulation diagnosis. International Review of Medical Sciences. 1994;6.

[19] Fehring RJ, Gaska N. Evaluation of the Lady Free Biotester in determining the fertile period. Contraception. 1998;57:325–8. 9673839 10.1016/s0010-7824(98)00039-0

[20] Biel Casals JM. Descripcion de un nuevo test de ovulacion y analisis de sus resultados. Medicina Clinica. 1968;50:385–92.

[21] Alagendran S, Archunan G, Achiraman SS. Prediction of Ovulation in Women through the Occurrence of Salivary Fern Prototype. The IUP Journal of Life Sciences. 2007:IJLS10708.

[22] Guida M, Barbato M, Bruno P, Lauro G, Lampariello C. Salivary ferning and the menstrual cycle in women. Clinical and Experimental Obstetrics & Gynecology. 1993;20:48–54. 8462188

[23] Pattanasuttinont S, Sereepapong W, Suwajanakorn S. The salivary ferning test and ovulation in clomiphene citrate-stimulated cycles. The Medical Association of Thailand. 2007;90:876–83. 17596040

[24] Guida M, Barbato M, Bruno P, Lauro G, Lampariello C. Salivary ferning and the menstrual cycle in women Clinical and experimental obstetrics & gynecology. 1993;20:48–54. 8462188

[25] Kullander S, Sonesson B. Studies on saliva in menstruating, pregnant and post-menopausal women. Acta Endocrinologica. 1965;48:329–36. 14259903 10.1530/acta.0.0480329

[26] Braat DD, Smeenk JM, Manger AP, Thomas CM, Veersema S, Merkus JM. Saliva test as ovulation predictor. The Lancet. 1998;352:1283–4. 10.1016/S0140-6736(05)70490-6 9788464

[27] Berardono B, Melani D, Ranaldi F, Giachetti E, Vanni P. Is the salivary "ferning" a reliable index of the fertile period? Acta Europaea Fertilitatis. 1993;24:61–5. 8171923

[28] Pardo-Carmona B, Moyano MR, Fernández-Palacinos R, Pérez-Marín CC. Saliva crystallisation as a means of determining optimal mating time in bitches Journal of Small Animal Practice. 2010;51:437–42. 10.1111/j.1748-5827.2010.00967.x 20670256

[29] Skalova I, Fedorova T, Brandlova K. Saliva Crystallization in Cattle: New Possibility for Early Pregnancy Diagnosis? Agricultura Tropica et Subtropica. 2013;46:102–4.

[30] Haberová T. A Preliminary Study of Saliva Crystallization in Bactrian Camels (*Camelus bactrianus*). In: Fernández Cusimani E, Havrland B, editors. 4th Scientific Conference of Institute of Tropics and Subtropics: Sustainable Use of Natural Resources in Tropics and Subtropics; Prague: CULS Prague; 2010. p. 28.

[31] Hodges K, Brown J, Heistermann M. Endocrine Monitoring of Reproduction and Stress In: Kleiman DG, Thompson KV, Baer CK, editors. Wild mammals in captivity: principles and techniques for zoo management. Chicago, IL, USA: University of Chicago Press; 2010 p. 447–68.

[32] Kutsukake N, Ikeda K, Honma S, Teramoto M, Mori Y, Haysaka I, et al Validation of Salivary Cortisol and Testosterone Assays in Chimpanzees by Liquid Chromatography-Tandem Mass Spectrometry. American Journal of Primatology. 2009;71:696–706. 10.1002/ajp.20708 19452511

[33] Wobber V, Hare B, Maboto J, Lipson S, Wrangham R, Ellison PT. Differential changes in steroid hormones before competition in bonobos and chimpanzees. PNAS. 2010;107:12457–62. 10.1073/pnas.1007411107 20616027 PMC2906573

[34] DiGiano L, Nagle CA, Quiroga S, Paul N, Farinati Z, Torres M, et al Salivary progesterone for the assessment of the ovarian function in the capuchin monkey (*Cebus apella*). International Journal of Primatology. 1992;13:113–23.

[35] Springer MS, Meredith RW, Gatesy J, Emerling CA, Park J, Rabosky DL, et al Macroevolutionary Dynamics and Historical Biogeography of Primate Diversification Inferred from a Species Supermatrix. PLoS ONE. 2012;7:e49521 10.1371/journal.pone.0049521 23166696 PMC3500307

[36] Wildman DE, Uddin M, Liu G, Grossman LI, Goodman M. Implications of natural selection in shaping 99.4% nonsynonymous DNA identity between humans and chimpanzees: Enlarging genus *Homo*. Proceedings of the National Academy of Sciences of the United States of America. 2003;100:7181–8. 12766228 10.1073/pnas.1232172100 PMC165850

[37] Fedorova T, Brandlová K, Bičíková M, Skálová I, Lukešová D. Salivary sex steroid hormones in female Bactrian camels (*Camelus bactrianus*) during different reproductive stages. Journal of Camel Practice and Research. 2015;22:61–6.

[38] Andonopoulos AP, Tzanakakis GN, Christophidou M. Light microscopy of dried saliva in the evaluation of xerostomia of the sicca syndrome. A preliminary report. Journal of Rheumatology. 1992;19:1390–2. 1433006

[39] Wildman DE, Uddin M, Liu G, Grossman LI, Goodman M. Implications of natural selection in shaping 99.4% nonsynonymous DNA identity between humans and chimpanzees: Enlarging genus Homo. Proceedings of the National Academy of Sciences of the United States of America. 2003;100:7181–8. 12766228 10.1073/pnas.1232172100 PMC165850

[40] Arévalo M, Sinai I, Jennings V. A fixed formula to define the fertile window of the menstrual cycle as the basis of a simple method of natural family planning. Contraception. 1999;60:357–60. 10715371 10.1016/s0010-7824(99)00106-7

[41] Behringer V, Stevens JMG, Hohmann G, Möstl E, Selzer D, Deschner T. Testing the Effect of Medical Positive Reinforcement Training on Salivary Cortisol Levels in Bonobos and Orangutans. PLoS ONE. 2014;9:e108664 10.1371/journal.pone.0108664 25250566 PMC4177400

[42] Knott CD, Emery Thompson M, Wich SA. The ecology of female reproduction in wild orangutans In: Wich SA, Atmoko SSU, Setia TM, van Schaik CP, editors. Orangutans: geographic variation in behavioral ecology and conservation New York: Oxford University Press; 2009 p. 171–88.

[43] Anderson HB, Thompson ME, Knott CD, Perlins L. Fertility and mortality patterns of captive Bornean and Sumatran orangutans: Is there a species difference in life history? Journal of Human Evolution. 2008;54:34–42. 17804037 10.1016/j.jhevol.2007.05.014

[44] Kuze N, Dellatore D, Banes GL, Pratje P, Tajima T, Russon AE. Factors affecting reproduction in rehabilitant female orangutans: young age at first birth and short inter-birth interval. Primates. 2012;53:181–92. 10.1007/s10329-011-0285-z 22109351

